# A Rare Case of Persistent Bacteremia Caused by Asaia spp. in an Infant

**DOI:** 10.7759/cureus.68577

**Published:** 2024-09-03

**Authors:** Athirah Mohammad, Nik Haszroel Hysham Nik Hashim

**Affiliations:** 1 Department of Medical Microbiology and Parasitology, Universiti Sains Malaysia School of Medical Sciences, Kota Bharu, MYS

**Keywords:** asaia, asaia siamensis, gram-negative bacteremia, 16s rrna detection test, catheter-related infections

## Abstract

*Asaia* spp. has recently been reported to cause opportunistic infections in humans and is becoming an emerging hospital pathogen. To our knowledge, this is the first report on *Asaia* spp. in Malaysia. bacteremia in an infant. A girl with underlying Hirschsprung's disease, who was on parenteral feeding via a central venous catheter, developed persistent multidrug-resistant Gram-negative bacteremia. Routine automated identification methods failed to identify the organism, which was later identified by 16S ribosomal RNA sequencing. Bacterial clearance was achieved after the removal of the catheter and initiation of IV amikacin. This case highlights the role of molecular identification and the clinical importance of *Asaia* spp. in causing infections in humans, especially in patients with indwelling devices.

## Introduction

*Asaia* is a genus of Gram-negative, acetic acid bacteria (AAB) in the family Acetobacteraceae, together with members of the genera *Acetobacter, Gluconobacter, and Gluconacetobacter* in the class of Alphaproteobacteria. Strains in this genus are characterized by weak or no ethanol oxidation to acetic acid. The genus *Asaia* was first characterized by Yamada et al. and initially included only one species, *Asaia bogorensis* [1]. Since then, the genus has expanded to include eight species: *A. bogorensis, A. lannensis* (previously known as *A. lannaensis*), *A. siamensis, A. krugthepensis, A. astilbis, A. platycodi, A. prunellae* and *A. spathodeae* [1-3]. These bacteria are known to be isolated from various tropical flowers, such as orchids and plumbago, and from fermented glutinous rice [4]. *Asaia* has also been found in the gut and reproductive tracts of mosquitoes from the genera *Aedes* and *Anopheles* [4]. As part of AAB, *Asaia* can survive in microoxic conditions, such as inside the gut of insects, through the use of ubiquinol oxidase [4]. They also have the ability to fix nitrogen, which benefits their mosquito hosts by supplying them with this essential nutrient [4]. This symbiotic relationship has raised an interest in this genus for malaria control because of its anti-*Plasmodium *property. The bacteria can even survive in acidic environments, even at a pH of 3 [5]. Early studies such as the one by Katsura et al. initially suggested that *A. bogorensis* had minimal impact on human health due to its inability to reproduce at the human body temperature of 37°C [6]. However, later studies, such as by Tuuminen et al., documented cases of bacteremia caused by *A. bogorensis* [7].

## Case presentation

A one-year-old girl with underlying Hirschsprung's disease and a history of multiple gastrointestinal surgeries and a perforated transverse colon was initially admitted to the surgical ward for an enterocutaneous fistula. She was significantly underweight, anemic, and had hypoalbuminemia. During her hospitalization, she developed loose stools and feeding intolerance. Empiric antibiotic therapy with IV cefotaxime, amikacin, and metronidazole was initiated to address suspected intra-abdominal sepsis. A primary abdominal ultrasound revealed no intra-abdominal collections. The patient responded well to conservative management of the enterocutaneous fistula and a 21-day course of antibiotics.

Due to challenging peripheral venous access and the need for total parenteral nutrition, a central jugular venous line (JVC) was inserted. On day 24 of hospitalization, she developed new-onset sepsis with provisional diagnoses of hospital-acquired pneumonia and catheter-related bloodstream infection (CRBSI). Septic workouts revealed an elevated total white cell count (TWC) and C-reactive protein (CRP) (Table 1).

**Table 1:** Routine laboratory investigations in the form of FBC parameters and CRP TWC: total white cell count, Hb: hemoglobin, CRP: C-reactive protein, FBC: full blood count

Parameters	Pre-admission	Day of admission				Normal range
		Day 1	Day 20	Day 24	Day 30	
TWC (10^9^/l)	8.21	10.5	13.76	14.76	9.5	Male: 3.80-9.70, female: 3.40-10.10
Hb (g/dl)	11.6	11.7	10.2	8.6	11.6	Male: 13.5-17.4, female: 11.6-15.1
Platelet (10^9^/l)	152	102	199	372	204	Male: 167-376, female: 158-410
CRP (mg/L)	38	-	-	133	109	<10

**Figure 1:**
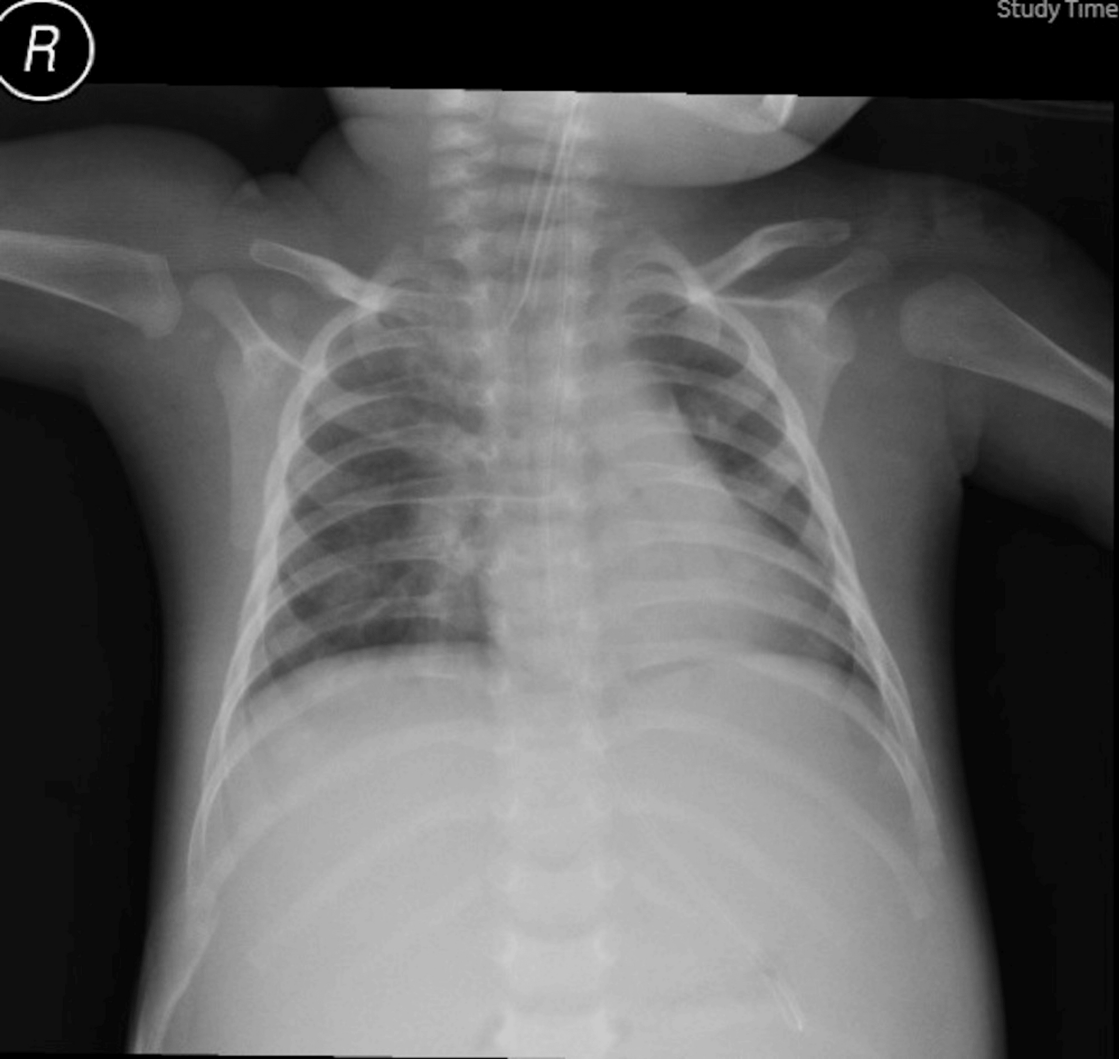
Upper lobe pneumonia and parapneumonic effusion shown in chest radiograph

A chest X-ray revealed right upper lobe pneumonia with parapneumonic effusion (Figure 1). Empirical treatment with IV imipenem, vancomycin, and metronidazole was started for suspected hospital-acquired infection and gastrointestinal sepsis. Blood cultures from both central and peripheral sites grew the same colony of Gram-negative bacilli with differential time to positivity, which was suggestive of CRBSI.

The patient continued to experience intermittent fever despite treatment with imipenem, metronidazole, and vancomycin. However, she was not in septic shock and remained hemodynamically stable on room air. The JVC line was removed, and a new femoral vein line was inserted.

**Figure 2:**
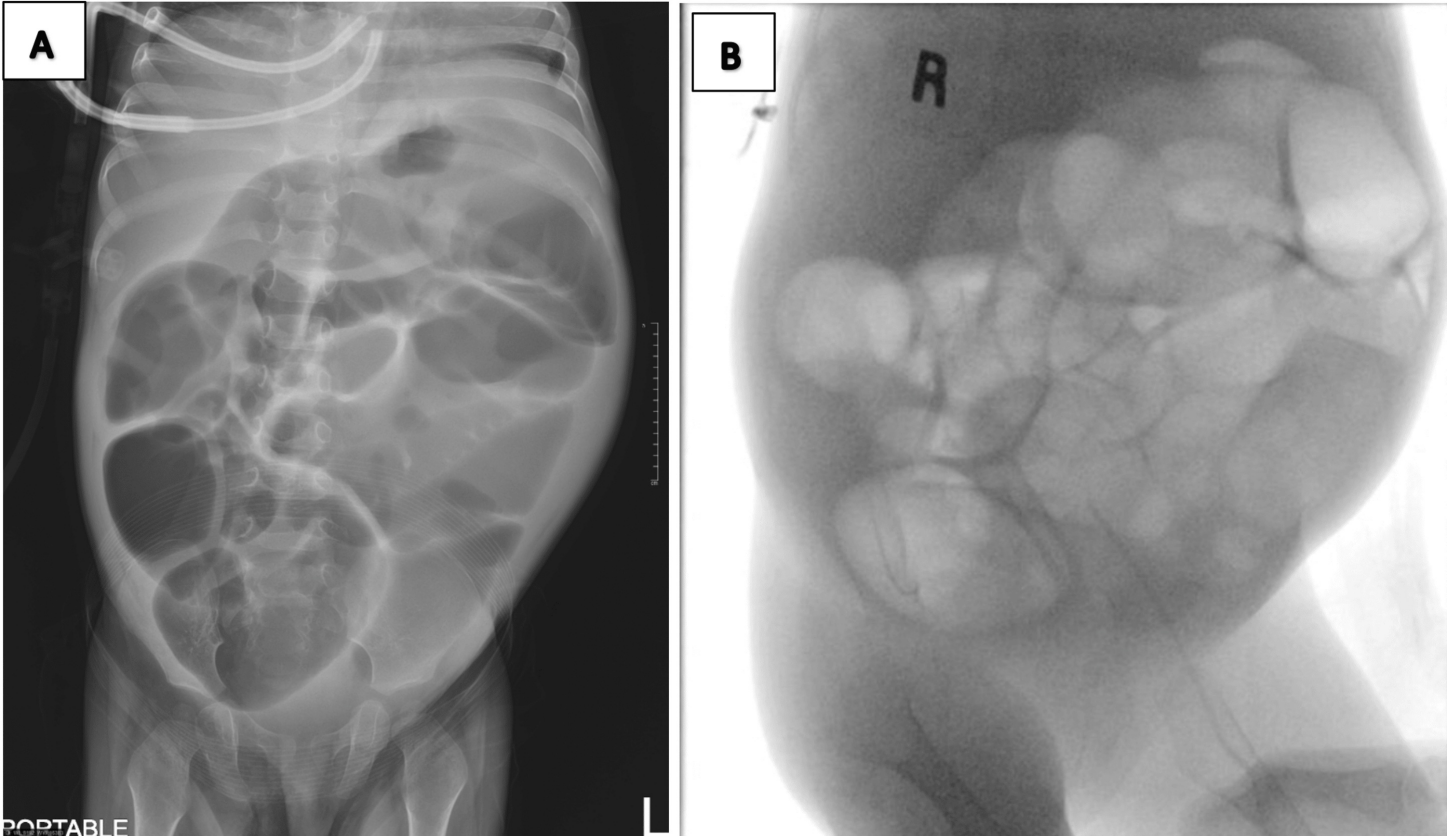
Abdominal radiograph (A) and lower gastrointestinal contrast study (B) Abdominal radiograph showed multiple bowel loop dilatation involving both central and peripheral, while no fluoroscopic evidence of contrast extravasation at the anastomotic site

**Figure 3:**
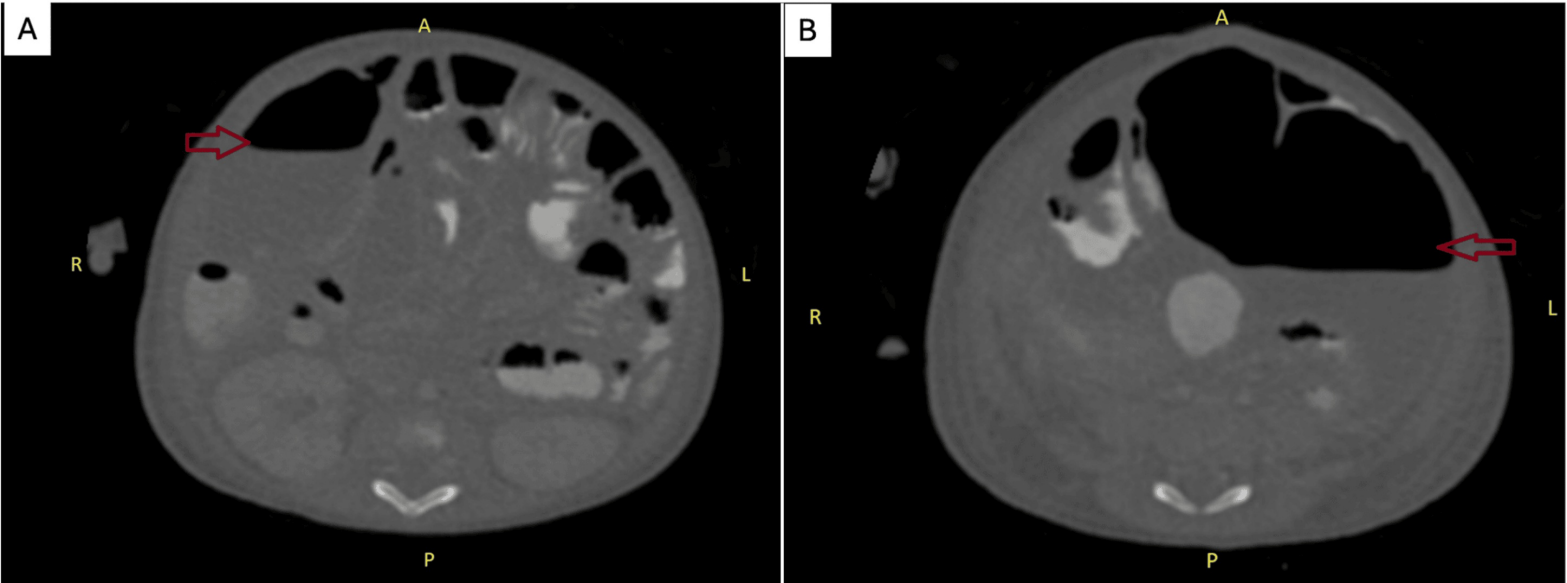
Multiple intra-abdominal collections with air-fluid levels at the right hypochondriac (A) and lower abdomen (B) marked with a red arrow, seen in follow-up CT abdomen with contrast CT: computer tomography

Her fever initially subsided within 24 hours after the removal of JVC, but her CRP level remained elevated. After a week of being afebrile, she developed a new spike in temperature. Subsequent blood cultures from both central and peripheral sites grew the same Gram-negative bacilli. At this point, the source of the bacteremia was still unknown. Notably, the culture of her parenteral feeding solution was not performed. A preliminary abdominal radiograph revealed multiple bowel loop dilatations involving both the central and peripheral. The lower gastrointestinal contrast study showed no fluoroscopic evidence of contrast extravasation at the anastomotic site to suggest leakage (Figure 2). Follow-up CT abdomen with contrast showed multiple intra-abdominal collections (Figure 3).

The femoral venous catheter was removed and replaced with a subclavian central venous Hickmann catheter. Six repeated aerobic blood cultures were sent and analyzed using the BACTEC FX system (Becton Dickinson, USA), which consistently yielded the same colony after an average incubation period of 27 hours.

IV amikacin was reinitiated as monotherapy. IV vancomycin was discontinued due to the absence of evidence for Gram-positive bacterial infection. The patient responded well to the new treatment regimen. Her fever resolved, and microbiological clearance was achieved with two subsequent negative blood cultures after three days of IV amikacin. The treatment was later switched to IV gentamicin, with a total duration of 14 days on IV aminoglycoside therapy. Additionally, the patient completed a one-week course of empirical IV fluconazole due to the risk of invasive candidiasis and persistent sepsis.

Microbiological method

**Figure 4:**
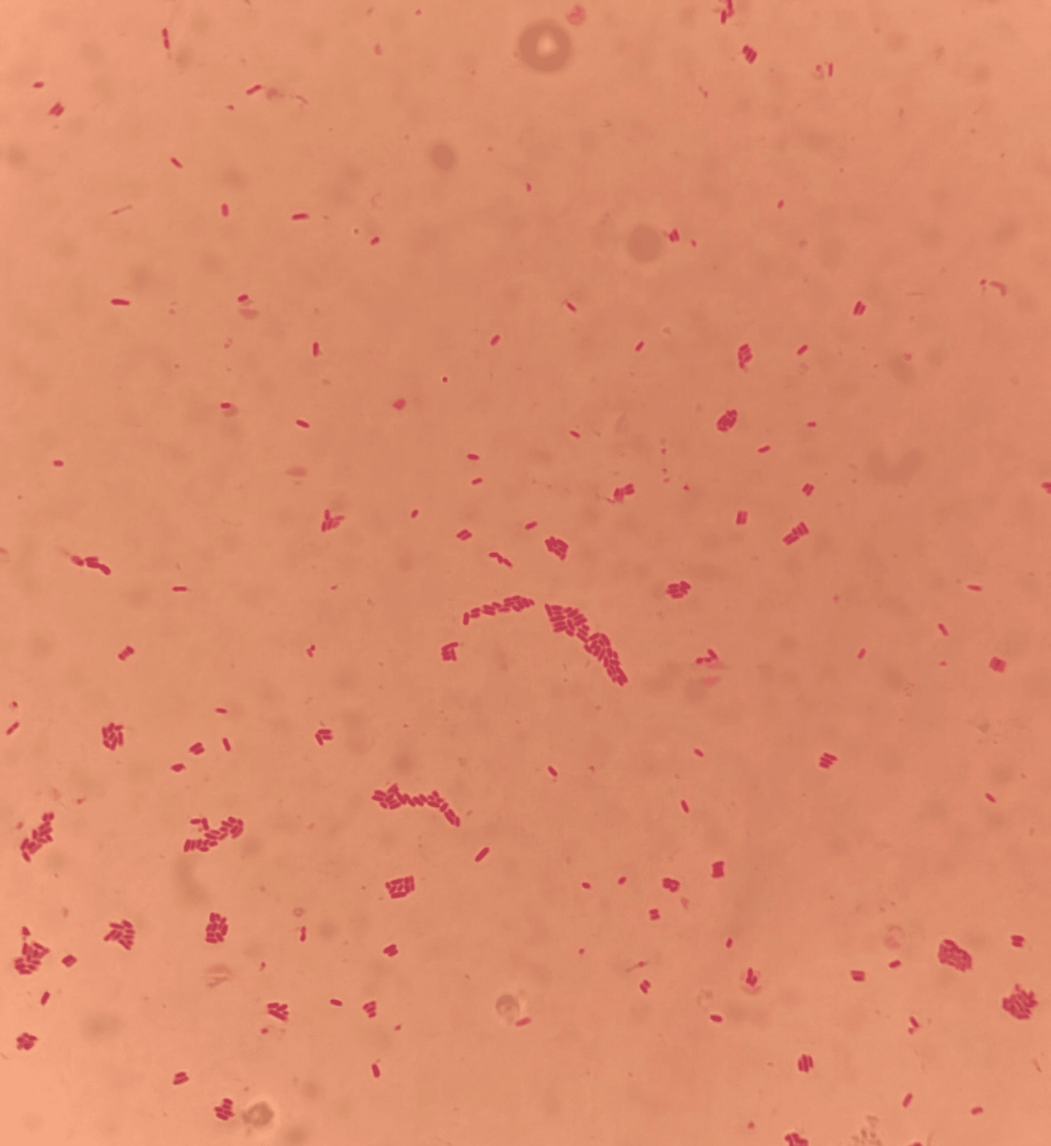
Gram-stain showed short, round Gram-negative bacilli

Microscopic examination from all the blood cultures revealed short, round, Gram-negative bacilli (Figure 4).

**Figure 5:**
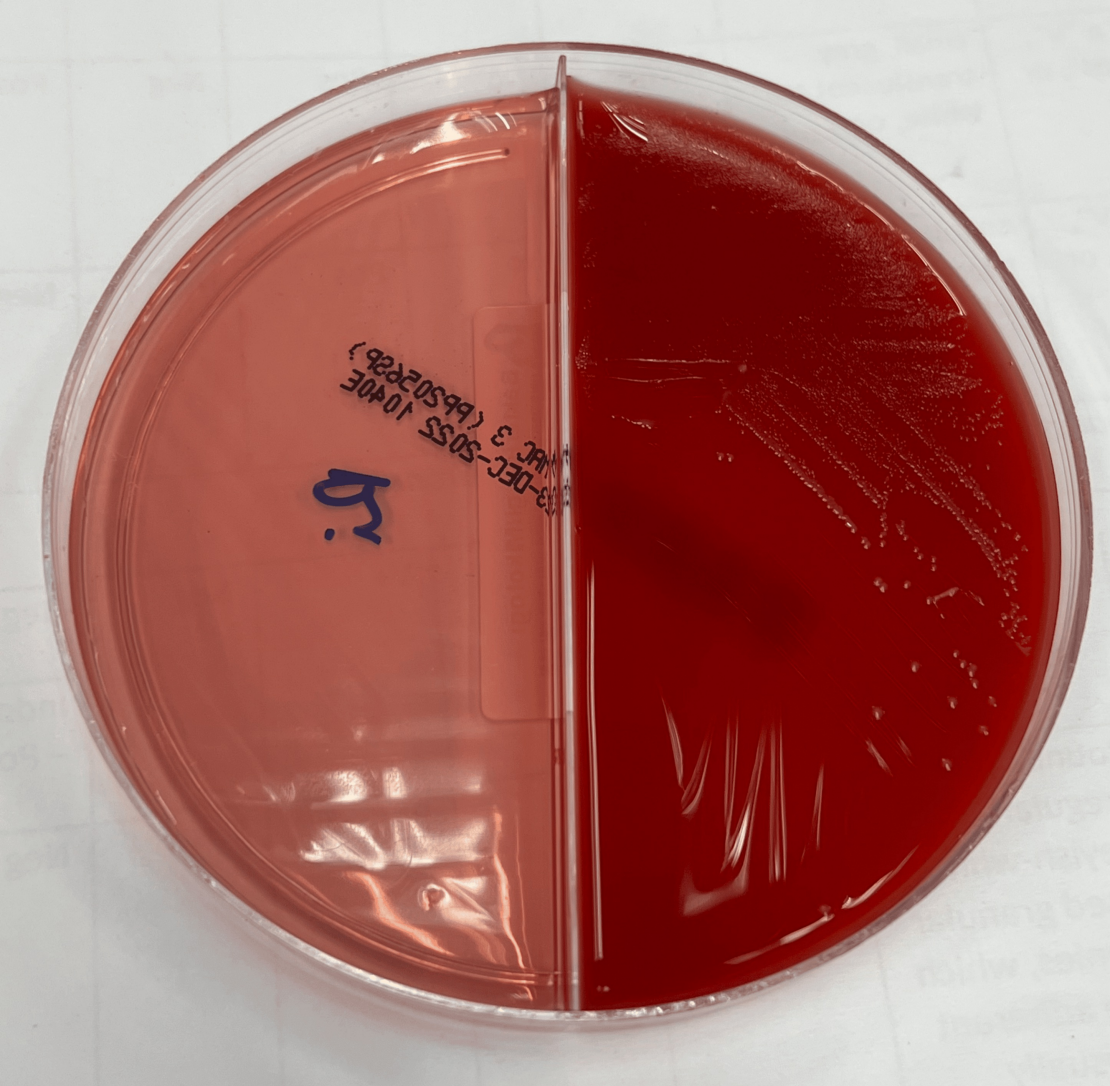
Growth of Asaia sp. on 5% sheep blood agar/MacConkey agar biplate incubated at 36°C in air after 48 hours

The blood sample was cultured on sheep blood agar and chocolate agar and incubated at 36°C in air and 5% CO2, respectively. The colonies on both sheep blood agar and chocolate agar were small, smooth, round, shiny, pale pink, and convex with an entire margin. There was no growth on MacConkey agar, even after extended incubation for 48 hours (Figure 5).

**Figure 6:**
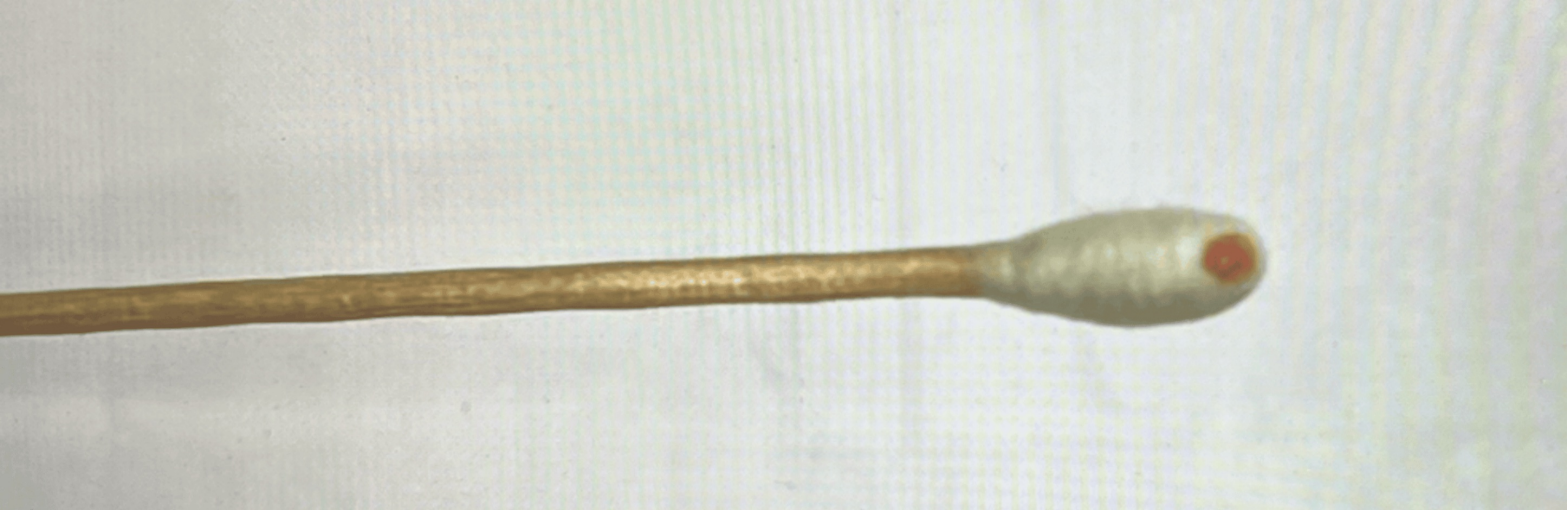
Pale pink pigmentation of the colonies after 48 hours of incubation

The colonies became more pigmented after 48 hours of incubation (Figure 6).

**Figure 7:**
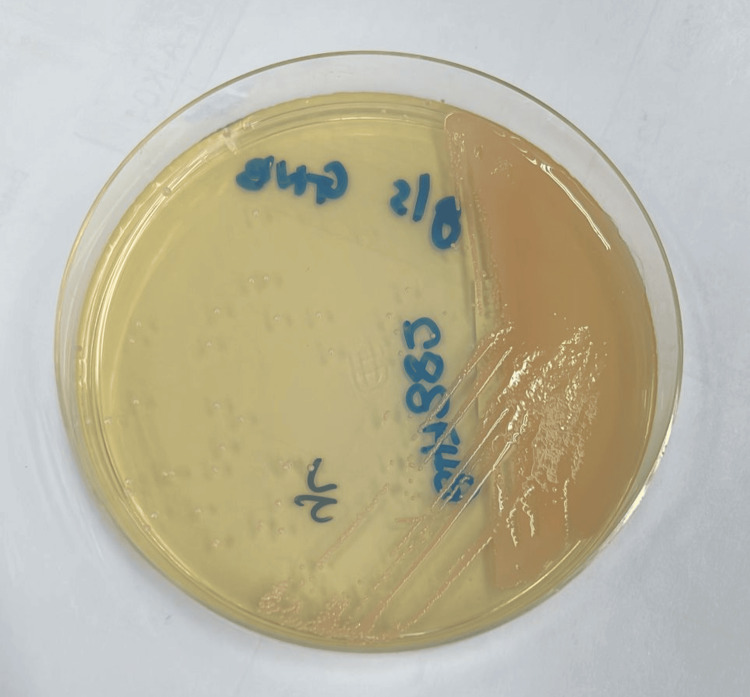
Asaia sp. colonies on SDA incubated at 30°C after 48 hours The appearance of *Asaia* colonies on the SDA as pink to colorless colonies is one of their unique morphological characteristics. SDA: Sabouraud Dextrose Agar

The blood sample was also cultured on Sabouraud Dextrose Agar (SDA) and incubated at 30°C for 48 hours to test for fungal growth. It grew the same colonies with more pronounced characteristics (Figure 7).

The organism was catalase-positive, oxidase-negative, and non-motile. Conventional biochemical tests showed negative reactions for indole production, urea hydrolysis, citrate hydrolysis, and methyl red test. Additionally, the triple sugar iron (TSI) test showed a neutral slant and butt with no H2S production.

The automated Vitek2™ (bioMérieux, Marcy-l'Étoile, France) GN card initially identified the organism as *Burkholderia cepacia* group with 94% probability and very good identification from the first isolate. However, the organism was identified as *Serratia fonticola* (99% probability, excellent identification) on the second isolate, despite having the same colony characteristics.

An initial antimicrobial susceptibility test (AST) was performed using the disc diffusion method (Kirby-Bauer) on Mueller-Hinton agar (MHA). The results showed no zone of inhibition for colistin, meropenem, ceftazidime, amoxicillin-clavulanate, piperacillin-tazobactam, trimethoprim-sulfamethoxazole, ciprofloxacin, and vancomycin. Only amikacin, gentamicin, and imipenem showed inhibition zones of 21 mm, 24 mm, and 15 mm, respectively.

Attempts to perform a minimum inhibitory concentration (MIC) test using an automated Vitek2™ AST card (intended for *Pseudomonas* spp.) were unsuccessful due to insufficient growth in the cartridge for analysis. A gradient MIC (E-test) was performed and indicated susceptibility to gentamicin according to the breakpoints proposed by the Clinical and Laboratory Standards Institute (CLSI) Interpretive Standards for the MIC for other non-Enterobacteriaceae. Other antimicrobials were not tested for MIC due to limited resources and knowledge of the organism at the time.

Unusual phenotypic antimicrobial resistance patterns and biochemical reactions raised concerns about the accuracy of the organism's identification. To address this, further molecular testing was performed using matrix-assisted laser desorption/ionization-time of flight (MALDI-TOF) technology, specifically the Microflex® (Billerica, MA, USA). This analysis yielded numerous organisms with unreliable identifications. The IVD-CE-approved software database used for diagnostic purposes in clinical microbiology laboratories showed the highest score of 1.26 for *Pseudomonas antarctica*. At this stage, it was suspected that the organism might not be included in the database of the automated identification system. Consequently, the colony was sent for genotypic identification through 16S ribosomal RNA (rRNA) gene sequencing (1st BASE, Singapore).

A 654 base pair 16S rRNA sequence obtained was then compared with those present in the GenBank database using the BLASTn program (National Center for Biotechnology Information, Bethesda, MD, USA). The taxonomy and the lineage reports were consistent with the genus *Asaia*, but different matches within the species of the genus were possible. The 16S rRNA result showed two distinct *Asaia* spp. with similarity of 100% (out of 654 bases), which were the genomes of *Asaia bogorensis* strain VN2013-60 (GenBank accession number: KX_449287.1), *Asaia bogorensis* strain NBRC 103486 (GenBank accession number: AB_682073.1), and* Asaia siamensis* strains LMG 21651 and S60-1 (GenBank accession numbers: MT_661481.1 and NR_024738.1, respectively). Further determination of species was not performed due to resource limitations.

## Discussion

Historically, *Asaia* spp. were known to cause spoilage in fruits and fruit beverages, and earlier observations indicated that these bacteria could not reproduce at 37°C. As a result, it was believed that *Asaia *spp. had minimal impact on human health [5,6]. However, in the past two decades, there have been a few reports of human infections, primarily transient bacteremia associated with *A. bogorensis* and *A. lannensis*. This has led to increased interest in their potential role as opportunistic pathogens and a re-evaluation of the methods used for identifying this genus [7-9]. To our knowledge, this is the first case of *Asaia* spp. causing significant nosocomial infection and persistent bacteremia reported in this region.

Reported cases of *Asaia* spp. include three instances of *A. bogorensis* infection in patients undergoing peritoneal dialysis, where the bacteria were associated with peritonitis. Additionally, two cases of *A. bogorensis* bacteremia were documented in IV drug users [7]. *A. lannensis* has been identified as the causative agent of nosocomial infections, including catheter-related infections in two pediatric patients with idiopathic dilated cardiomyopathy who had indwelling peripherally inserted central catheters [9]. Another case involved an *A. lannensis* infection in a patient who had undergone a hematopoietic stem cell transplant, with a central venous catheter as the likely source of infection. More recently, *A. lannensis* bacteremia was reported in a psychiatric patient who self-injected unsafe water. Additionally, a case of *Asaia* spp. was isolated from the sputum of a two-year-old child with cystic fibrosis (CF), suggesting possible colonization of AAB in CF patients. However, species-level identification was inconclusive despite various molecular approaches [8]. Given these identification challenges, the true prevalence of *Asaia* spp. infections in humans may be underestimated.

The role of *Asaia* spp. as a human pathogen remains unclear. A study aimed at detecting *Asaia* spp. in a healthy population by analyzing both *Asaia* spp. DNA and antibodies in the whole blood and serum of 496 individuals exposed to mosquito bites found negative results for both *Asaia* spp. DNA and antibodies [10]. This suggests *Asaia* spp. have a very low pathogenic potential. In the cases described above, bacteremia caused by *Asaia* spp. was generally transient, with bacteremic clearance achieved either through effective antimicrobial treatment or removal of a known infection source, such as a catheter tip. Clinical improvement is typically followed promptly.

In the current case, the persistence of *Asaia spp. *bacteremia lasted approximately two weeks, likely due to delays in source removal and the initiation of appropriate antimicrobial therapy. The patient’s fever resolved immediately following the removal of the catheter and the start of IV amikacin. The stability of the patient, despite being immunocompromised (very young age, underweight, anemic, and hypoalbuminemic), suggests that this isolate has low virulence.

In terms of infection control, delays in identification and unfamiliarity with this organism hindered a thorough investigation of other potential infection sources, such as cultures from the infusate, infusion pump, or antiseptic solutions. Previous reports and the present case indicate that common risk factors for *Asaia *spp. infections include the presence of indwelling devices, invasive procedures, or skin breaches. Additionally, a prolonged history of broad-spectrum antimicrobial use may predispose individuals to *Asaia *spp. infections.

Based on previous cases of infection with *Asaia* spp., phenotypic methods have not reliably identified this organism. Phenotypic tests used for the rapid identification of AAB can exhibit variability, primarily due to environmental factors such as culture conditions [11]. *Asaia* spp. typically produce small (1-2 mm in diameter), non-hemolytic, pale-pigmented colonies on blood and chocolate agar but are frequently not detected on MacConkey agar. Biochemically, *Asaia* spp. are strongly saccharolytic, rapidly acidifying glucose, mannitol, xylose, and L-arabinose. They are catalase-positive, oxidase-negative, indole and urea-negative, and non-motile.

In this case, the isolate exhibited inert reactions at 36°C. The optimal growth temperature for *Asaia* spp. ranges from 22°C to 30°C, with a decreased growth rate at 37°C and no growth at 42°C, as demonstrated here. Literature suggests a scheme using conventional methods to differentiate *Asaia* spp. from other pink-pigmented non-fermentative Gram-negative organisms, such as *Azospirillum, Roseomonas*, and *Methylobacterium*. However, our isolate exhibited some confounding characteristics. There were no reactions on TSI agar, and motility was not observed. Additionally, our isolate failed to grow on MacConkey agar at 48 hours, consistent with reports of variable growth on this medium. Some literature indicates that growth on MacConkey agar may be observed only after 72 hours of incubation [9]. This supports the notion that *Asaia* spp. colony characteristics can be influenced by cultural conditions, and phenotypic methods may not be entirely reliable for their identification.

The atypical characteristics of this isolate raised concerns about potential misidentification, despite accurate identification by the Vitek2™ system, which identified it as *Burkholderia cepacia* complex and *Serratia fonticola*. *Asaia *spp. are not included in the commercially available identification system databases that rely on growth-based technology [9]. The MALDI-TOF system also faced difficulties in identifying this organism, as evidenced by its failure to accurately identify *A. lannensis* in current and previous cases [3]. This situation indicates that the database for this genus is inadequate and highlights the need for a larger number of reference strains to update and improve the identification system.

Final identification of *Asaia* spp. in all the reported cases required 16S rRNA gene sequence analysis [3,7-9]. However, distinguishing *Asaia* spp. at the species level can be challenging due to the variable length of the sequences obtained with different 16S rRNA primers, which often necessitates additional testing such as restriction fragment length polymorphism of the 16S rRNA gene [2,3]. In our case, the 654 base pair 16S rRNA sequence showed 100% similarity with *Asaia siamensis* and *Asaia bogorensis*. *A. siamensis* shares morphological, physiological, and biochemical characteristics with *A. bogorensis*, except for its inability to produce acid from dulcitol and slightly lower DNA G+C content. To our knowledge, human infections caused by *A. siamensis* have not been reported since its introduction in 2001. We did not perform the discriminatory dulcitol tests or additional molecular tests to differentiate between these two species due to their unavailability in our laboratory and the shorter length of our 16S rRNA sequence compared to the >1400 base pair sequences reported previously [2,3,6].

No established AST guidelines are available for *Asaia* spp. due to their rarity in causing human infections. Initial AST (disc diffusion) for this isolate was performed using available resources to assist clinicians in selecting appropriate antimicrobial therapy. However, no results were obtained with the automated Vitek2™ AST Gram-negative panel for this strain, largely due to insufficient growth [3,9]. Consequently, gradient MICs were deemed the most suitable AST method for this organism. The optimal agar and incubation conditions for *Asaia *spp. need further investigation. Previous reports for *A. bogorensis *and *A. lannensis* utilized Mueller-Hinton blood agar supplemented for AST testing, with the optimal incubation temperature for *Asaia *spp. proposed to be 30°C [8]. In this case, AST was conducted at 36°C using MHA and evaluated after 20-24 hours, as this isolate could grow under these conditions.

A high level of resistance has been documented in *Asaia* spp. isolates. This organism exhibits resistance to all beta-lactams and some beta-lactam/beta-lactamase inhibitor combinations, including ceftazidime, cefepime, piperacillin/tazobactam, and carbapenems, except for imipenem. Notably, imipenem is the only carbapenem with in vitro activity against *Asaia lannensis* [9]. In our case, the disk diffusion method revealed a zone of inhibition of 15 mm for imipenem, although the MIC was not determined. The patient received imipenem as empirical therapy. *Asaia* spp. also shows resistance to trimethoprim, amikacin, vancomycin, aztreonam, penicillin, and ampicillin, according to CLSI MIC breakpoints for non-Enterobacterales [3,9]. Aminoglycosides and tetracyclines were identified as the only effective antimicrobials against *Asaia *spp. [3,7-9]. Consistent with previous reports, the strain was susceptible only to aminoglycosides, as demonstrated by both disk diffusion and E-test methods.

## Conclusions

This is the first report of *A. siamensis* causing human infection. Our 654 base pair 16s rRNA sequence showed 100% similarity with two *Asaia spp*. (*A. siamensis and A. bongorensis*), which both shared similar characteristics except for the inability of* A. siamensis *to produce acid from dulcitol and slightly lower DNA G+C contents. Clinicians and microbiologists should be aware of the role of *Asaia* spp. as an opportunistic pathogen in immunosuppressed pediatric patients and infections of indwelling devices. This case highlights the possibility of misidentification of *Asaia* spp. by automated systems used in routine clinical laboratories, which may hinder appropriate and timely intervention of bacteremia, as well as emphasizes the importance of quicker molecular identification for these unusual microorganisms. The organism was found resistant toward most beta-lactams and some beta-lactam/beta-lactamase inhibitor combinations; further analysis of the mechanism of resistance is required.

## References

[1] Yamada Y, Katsura K, Kawasaki H (2000). Asaia bogorensis gen. nov., sp. nov., an unusual acetic acid bacterium in the alpha-Proteobacteria. Int J Syst Evol Microbiol.

[2] Malimas T, Yukphan P, Takahashi M (2008). Asaia lannaensis sp. nov., a new acetic acid bacterium in the Alphaproteobacteria. Biosci Biotechnol Biochem.

[3] Carretto E, Visiello R, Bardaro M, Schivazappa S, Vailati F, Farina C, Barbarini D (2016). Asaia lannensis bacteremia in a 'needle freak' patient. Future Microbiol.

[4] Chouaia B, Rossi P, Epis S (2012). Delayed larval development in Anopheles mosquitoes deprived of Asaia bacterial symbionts. BMC Microbiol.

[5] Horsáková I, Voldřich M, Čeřovský M, Sedláčková P, Šicnerová P, Ulbrich P (2009). Asaia sp. as a bacterium decaying the packaged still fruit beverages. Czech J Food Sci.

[6] Katsura K, Kawasaki H, Potacharoen W (2001). Asaia siamensis sp. nov., an acetic acid bacterium in the alpha-proteobacteria. Int J Syst Evol Microbiol.

[7] Tuuminen T, Heinäsmäki T, Kerttula T (2006). First report of bacteremia by Asaia bogorensis, in a patient with a history of intravenous-drug abuse. J Clin Microbiol.

[8] Alauzet C, Teyssier C, Jumas-Bilak E (2010). Gluconobacter as well as Asaia species, newly emerging opportunistic human pathogens among acetic acid bacteria. J Clin Microbiol.

[9] Juretschko S, Beavers-May TK, Stovall SH (2010). Nosocomial infection with Asaia lannensis in two paediatric patients with idiopathic dilated cardiomyopathy. J Med Microbiol.

[10] Epis S, Gaibani P, Ulissi U (2012). Do mosquito-associated bacteria of the genus Asaia circulate in humans?. Eur J Clin Microbiol Infect Dis.

[11] Trček J, Barja F (2015). Updates on quick identification of acetic acid bacteria with a focus on the 16S-23S rRNA gene internal transcribed spacer and the analysis of cell proteins by MALDI-TOF mass spectrometry. Int J Food Microbiol.

